# Termite-Susceptible Species of Wood for Inclusion as a Reference in Indonesian Standardized Laboratory Testing

**DOI:** 10.3390/insects3020396

**Published:** 2012-03-28

**Authors:** Arinana, Kunio Tsunoda, Elis N. Herliyana, Yusuf S. Hadi

**Affiliations:** 1 Faculty of Forestry, Bogor Agricultural University, Bogor 16680, West Java, Indonesia; E-Mails: elisherliana@yahoo.com (E.N.H); yshadi@indo.net.id (Y.S.H); 2 Research Institute for Sustainable Humanosphere, Kyoto University, Uji 611-0011 Kyoto, Japan

**Keywords:** percent mass loss, termite mortality, wood feeding rate, *Coptotermes formosanus*, SNI 01.7207-2006, JIS K 1571-2004

## Abstract

Standardized laboratory testing of wood and wood-based products against subterranean termites in Indonesia (SNI 01.7207-2006) (SNI) has no requirement for the inclusion of a comparative reference species of wood (reference control). This is considered a weakness of the Indonesian standard. Consequently, a study was undertaken to identify a suitable Indonesian species of community wood that could be used as a reference control. Four candidate species of community woods: *Acacia mangium*, *Hevea brasiliensis*, *Paraserianthes falcataria* and *Pinus merkusii* were selected for testing their susceptibility to feeding by *Coptotermes formosanus*. Two testing methods (SNI and the Japanese standard method JIS K 1571-2004) were used to compare the susceptibility of each species of wood. Included in the study was *Cryptomeria japonica*, the reference control specified in the Japanese standard. The results of the study indicated that *P. merkusii* is a suitable reference species of wood for inclusion in laboratory tests against subterranean termites, conducted in accordance with the Indonesian standard (SNI 01.7207-2006).

## 1. Introduction

Indonesia has extensive community forests which produce a diverse range of wood species. In these forests, four species of wood are particularly abundant in both quantity and future supply: *Acacia mangium*, *Paraserianthes falcataria*, *Hevea brasiliensis* and *Pinus merkusii*. However, most of the wood species from the community forests are highly vulnerable to wood-destroying organisms such as insects and fungal decay. Of the more than 4,000 wood species in Indonesia, most of them (80–85%) are regarded as poor quality due to their low natural durability. Furthermore, there is a profound lack of knowledge on their characteristics and uses [1].

Determination of the resistance of wood and wood-based materials to damage by termites is largely dependent on well-designed and well-executed laboratory evaluations. It is therefore important that the experimental design of such laboratory tests include a suitable reference material (control). This enables the researcher to compare the performance of the candidate material/s under test with that of a known reference control. Furthermore, a reference control can often be used to monitor the viability and vigor of the test termites used in the laboratory test. The inclusion of a suitable reference control in the laboratory test can also be used to compare mortality of termites with those that are exposed to the candidate materials. Consequently, the choice of an appropriate wood species to serve as a reference control is most important for the conduct of comparative laboratory tests on the termite-resistance of both untreated and preservative-treated woods.

Unfortunately, the Indonesian standard SNI 01.7207-2006 does not specify the use of a selected reference control that will enable comparison of data obtained by different researchers. Thus, the aim of our study is to identify a suitable wood species for inclusion as a reference control in the standard.

## 2. Experimental Section

### 2.1. Wood Species

The candidate wood species used in this study were *Acacia mangium*, *Paraserianthes falcataria*, *Hevea brasiliensis* and *Pinus merkusii*. All four species are rated as durability classes III and IV (based SNI 01.7207-2006 [2]. *Cryptomeria japonica* was selected as the comparative reference control species as it is used as such in the Japanese Standard JIS K1571-2004 [3]. All five wood species were subjected to bioassay against termites according to the test methods described in SNI 01.7207-2006 and Japanese Standard JIS K1571-2004 (recently revised as JIS K1571-2010).

### 2.2. Test Method According to SNI 01. 7207-2006

**Figure 1:**
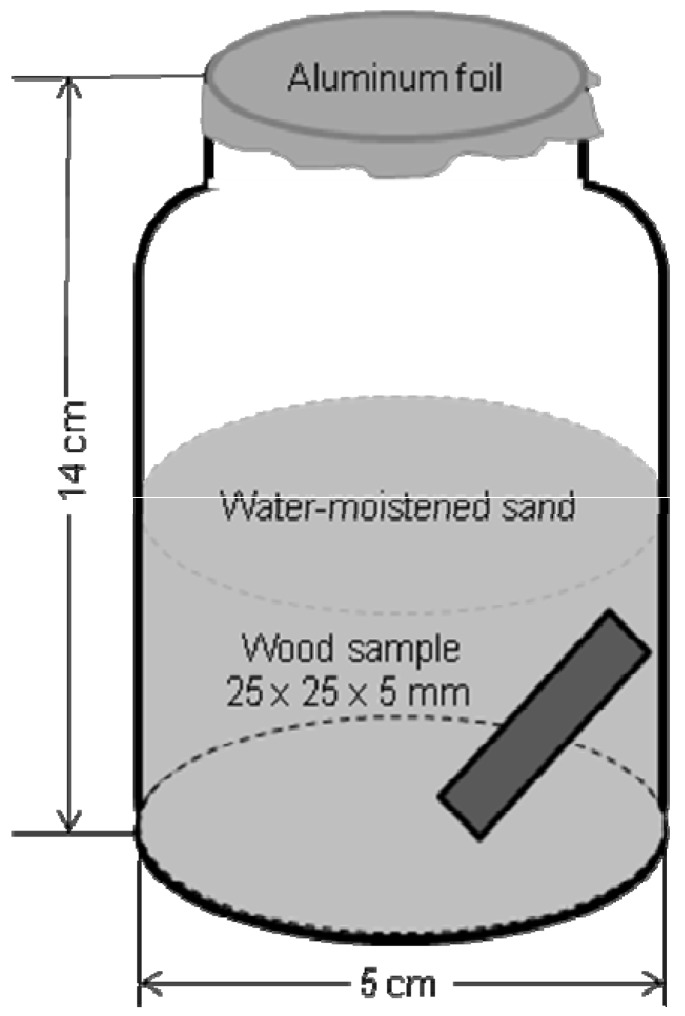
Diagram of the Indonesian Standard SNI 01.7207-2006 test method with termites.

This standard describes a 4-week, no choice laboratory test using 200 g of sand matrix, 50 mL of distilled water and 200 workers of *Coptotermes formosanus* Shiraki*.* Test specimens (25 × 25 × 5 mm) were placed upright position within a glass jar one of the 25 mm edges resting on the inside wall of the jar. We performed this research in five replicates. Full details of the test method are given in SNI 01.7207-2006 and [4]. A diagram of the test method according to SNI 01.7207-2006 is provided in Figure 1.

### 2.3. Test Method According to JIS K1571-2004 (Recently Revised as JIS K1571-2010)

**Figure 2:**
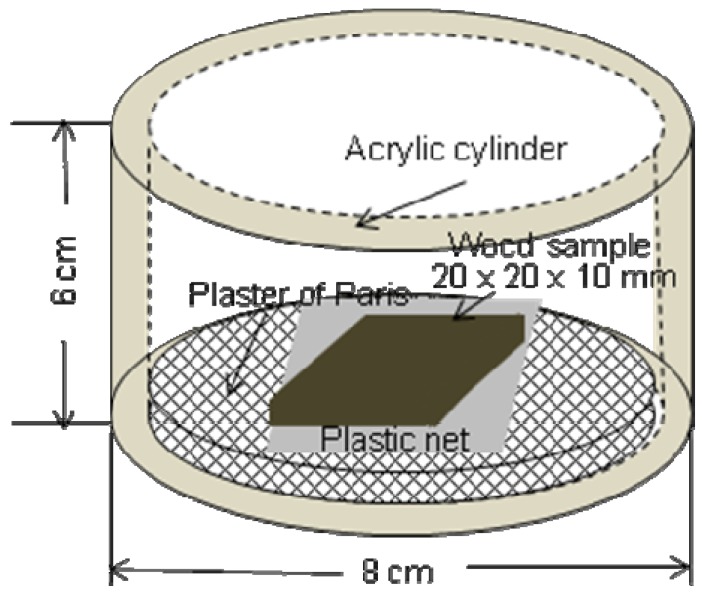
Diagram of the Japanese standard JIS K 1571-2004 test method with termites.

This laboratory test method is similar to that described in the Indonesian standard. The Japanese method is also a no-choice test. Sugi (*Cryptomeria japonica*) sapwood specimens are used as the untreated reference wood species (control). This is implied by the no-choice description (Figure 2). The test specimen is placed on a plastic net to avoid direct contact with the moistened layer of Plaster of Paris on the base of a cylindrical acrylic container. *C full. formosanus* (150 workers and 15 soldiers) were added to each test container. Full details of the test method are given in JIS K1571-2004*.* A diagram of the test method according to JIS K1571-2004 is provided in Figure 2. Test containers containing termites were maintained at 28 ± 2 °C and 80% RH for three weeks in the dark.

### 2.4. Calculation of Results

Percent mass loss of the individual wood specimen is calculated by the difference between the before and after weights according to the following equation:

     Percent mass loss = (W_1_ − W_2_)/W_1_ × 100

where W_1_ = weight of oven-dried wood specimen before test (g), W_2_ = weight of oven-dried wood specimen after test (g). When the mean percent mass loss of five untreated wood specimens is <15%, the test is not valid and should be repeated.

In addition to the percent mass loss, termite mortality was calculated according to the following equations: 

  Termite mortality (%) for SNI = (number of dead workers)/200 × 100

  Termite mortality (%) for JIS = (number of dead workers)/150 × 100

The feeding (wood consumption) rates are most useful for comparing test results obtained with wood species of different densities. In order to calculate the feeding rate, an assumption is made that termites die linearly with time. This statement was also confirmed by Su and LaFarge 1984 [5].

On the basis of the above assumption, feeding rates were calculated according to the following equation:

Feeding rate (mg/termite/week) = (weight of wood eaten by termites)/termites × test period (weeks).

## 3. Results and Discussion

### 3.1. Results of Laboratory Test According to SNI 01. 7202-2006

Data on mean mass loss, mean termite mortality and wood consumption obtained from the laboratory test conducted according to the Indonesian Standard (SNI) are presented in Table 1.

**Table 1:** Data on mean mass loss, mortality and wood consumption rates at the conclusion of the SNI laboratory test.

Wood Species	Mass Loss (%)	Termite Mortality (%)	Wood Consumption (µg/termite/day)
*Acacia mangium*	11.6 ± 1.24	27 ± 4.0	43 ± 4.5
*Hevea brasiliensis*	21.0 ± 1.05	22 ± 5.0	79 ± 4.8
*Paraserianthes falcataria*	24.5 ± 1.76	23 ± 3.5	49 ± 4.0
*Pinus merkusii*	25.4 ± 3.35	9 ± 4.8	79 ± 9.9
*Cryptomeria japonica*	42.1 ± 1.91	19 ± 5.2	82 ± 5.6

Mean mass losses ranged from 11.6% (*A.* *mangium*) to 42.1% (*C.* *japonica*). One of the requirements of the Japanese standard method of test is that the untreated control shall sustain a mean mass loss of more than 15% for the test to be considered valid. If this occurs, the test is deemed valid. Given the Japanese requirement, the results of the Indonesian SNI test suggest that *H.* *brasiliensis*, *P.* *falcataria* and *P.* *merkusii* (mean mass losses of 21.0%, 24.5%, and 25.4%, respectively) could be suitable candidates for reference controls. *A.* *mangium* with a mean mass loss of 11.6% failed to qualify as a suitable reference control. It appears that under the conditions of the SNI test, *A.* *mangium* displayed some resistance to attack by *C. formosanus*, probably due to its inherent extractive content.

### 3.2. Results of Laboratory Test According to JIS K 1571-2004

Data on mean mass loss (%), mean mortality (%) and wood consumption obtained from the laboratory test conducted according to the Japanese Standard (JIS) are presented in Table 2. The mortality results obtained from the JIS and SNI termite tests both had similar trends.

**Table 2:** Data on mean mass loss, mortality and wood consumption rates at the conclusion of the JIS laboratory test.

Wood Species	Mass Loss (%)	Termite Mortality (%)	Wood Consumption (µg/termite/day)
*Acacia mangium*	6.1 ± 1.19	32 ± 6.1	55 ± 8.7
*Hevea brasiliensis*	15.8 ± 1.29	8 ± 3.8	129 ± 10.0
*Paraserianthes falcataria*	14.2 ± 2.23	11 ± 3.6	66 ± 6.5
*Pinus merkusii*	16.8 ± 3.29	7 ± 1.9	95 ± 21.1
*Cryptomeria japonica*	21.8 ± 2.33	12 ± 4.0	98 ± 10.6

Mean wood consumption per termite per day ranged from 55 µg (*A. mangium*) to 129 µg (*H. brasiliensis*). Mean wood consumption per termite per day for *P.* *falcataria*, *P. merkusii* and *C. japonica* was 66 µg, 95 µg and 98 µg, respectively. Like the SNI results, mean wood consumption of *P. merkusii* was also quite high (95 µg).

## 4. Conclusions

The results of this study suggest that *P. merkusii*, *P. falcataria* or *H. brasiliensis* could be suitable reference species of wood (comparative reference controls) for inclusion in laboratory tests against subterranean termites, conducted in accordance with the Indonesian standard SNI 01.7207-2006. The use of such reference species of wood will allow more data to be obtained on the viability and vigor of the termites being used in tests conducted on the durability of wood and wood products against subterranean termites in Indonesia. Furthermore, the inclusion of a reference wood species will enable comparison of data obtained by different researchers.
